# Social determinants and access to health services in patients with COVID-19: a cross-sectional study^*^

**DOI:** 10.1590/1980-220X-REEUSP-2023-0324en

**Published:** 2024-03-08

**Authors:** Maria Williany Silva Ventura, Francisca Elisângela Teixeira Lima, Paula dos Santos Brito, Lívia Maia Pascoal, Nila Larisse Silva de Albuquerque, Paulo César de Almeida

**Affiliations:** 1 Universidade Federal do Ceará, Faculdade de Farmácia, Odontologia e Enfermagem, Departamento de enfermagem, Fortaleza, CE, Brazil.; 2 Universidade Federal do Maranhão, Departamento de Enfermagem, Imperatriz, MA, Brazil.; 3 Universidade da Integração Internacional da Lusofonia Afro-Brasileira, Departamento de Enfermagem, Redenção, CE, Brazil.; 4 Universidade Estadual do Ceará, Departamento de Enfermagem, Fortaleza, CE, Brazil.

**Keywords:** Social Determinants of Health, COVID-19, Access to Health Services, Facilities and Services Utilization, Determinantes Sociales de la Salud, COVID-19, Accesibilidad a los Servicios de Salud, Utilización de Instalaciones y Servicios, Determinantes Sociais da Saúde, COVID-19, Acesso aos Serviços de Saúde, Utilização de Instalações e Serviços

## Abstract

**Objective:**

To verify the association between social determinants of health and access to health services for COVID-19 patients.

**Method:**

Analytical, cross-sectional study, carried out in three states in the Northeast of Brazil (Ceará, Maranhão and Pernambuco), with 968 patients, using questionnaires with sociodemographic data, determinants and the Primary Care Assessment Tool, adapted to the reality of COVID-19, with 58 items, classified as high (score ≥ 6.6) and low (score < 6.6), whose high value reveals better standards of access to health services. The Chi-square test was used for comparative analysis.

**Results:**

There was a significant difference (p < 0.05) between the domains of the instrument and the following determinants: age, skin color, body mass index, origin, schooling, employment, services close to home, first service, income and means of transport.

**Conclusion:**

Access to health services for people with COVID-19 was associated with various determinants, including individual, behavioural and social ones, correlated with the structural and organizational aspects of the health services offered by the three states of Northeastern Brazil.

## RESUMO

**Objetivo:**

Verificar a associação dos determinantes sociais da saúde com o acesso de pacientes com COVID-19 aos serviços de saúde.

**Método:**

Estudo analítico, transversal, desenvolvido em três estados do Nordeste brasileiro (Ceará, Maranhão e Pernambuco), com 968 pacientes, utilizando-se de questionários de dados sociodemográficos, determinantes e do *Primary Care Assessment Tool*, adaptado para realidade da COVID-19, com 58 itens, classificado em alto (escore ≥ 6,6) e baixo (escore < 6,6), cujo valor alto revela melhores padrões de acesso aos serviços de saúde. Para análise comparativa, empregou-se o teste do Qui-quadrado.

**Resultados:**

Verificou-se diferença significativa (p < 0,05) entre os domínios do instrumento e os seguintes determinantes: idade, cor da pele, índice de massa corporal, procedência, escolaridade, vínculo empregatício, serviços próximos à residência, primeiro serviço de atendimento, renda e meios de transporte.

**Conclusão:**

O acesso aos serviços de saúde de pessoas com COVID-19 esteve associado aos diversos determinantes, sendo estes individuais, comportamentais, sociais, correlacionados aos aspectos estruturais e organizacionais dos serviços de saúde ofertados pelos três estados do Nordeste brasileiro.

## RESUMEN

**Objetivo:**

Verificar la asociación de los determinantes sociales de la salud con el acceso de los pacientes con COVID-19 a los servicios de salud.

**Método:**

Estudio analítico, transversal, realizado en tres estados del Nordeste de Brasil (Ceará, Maranhão y Pernambuco), con 968 pacientes, utilizando cuestionarios con datos sociodemográficos, determinantes y la Herramienta de Evaluación de la Atención Primaria (PCATool), adaptada a la realidad de la COVID-19, con 58 ítems, clasificados en alto (puntuación ≥ 6,6) y bajo (puntuación < 6,6), cuyo valor alto revela mejores estándares de acceso a los servicios de salud. Se utilizó la prueba de chi-cuadrado para analizar las comparaciones.

**Resultados:**

Hubo diferencia significativa (p < 0,05) entre los dominios del instrumento y los siguientes determinantes: edad, color de piel, índice de masa corporal, origen, escolaridad, empleo, servicios cercanos al domicilio, primer servicio, ingreso y medio de transporte.

**Conclusión:**

El acceso a los servicios de salud de las personas con COVID-19 se asoció a diversos determinantes, entre ellos individuales, comportamentales y sociales, correlacionados con los aspectos estructurales y organizativos de los servicios de salud ofrecidos por los tres estados del nordeste de Brasil.

## INTRODUCTION

A new disease with an unidentified etiological agent was detected in December 2019 at the city of Wuhan, China^(1)^. The disease, later named COVID-19, quickly spread to other countries, becoming a global health problem^(2)^. Over the course of 2020, the number of cases grew, generating large proportions on all continents, reaching 66 million infected people worldwide, in the same period, Brazil reached a number of more than 6 million infected people and more than 176,000 deaths from the disease^(3)^. In December 2023, COVID-19 exceeded 770 million confirmed cases worldwide^(4)^.

In this context, the difficulties faced by health institutions throughout the COVID-19 pandemic have been numerous, such as increased demand, a change in the care profile, a lack of supplies, untrained professionals, inadequate funding and growing pent-up demand^(5)^.

Early testing, sustained and affordable access to health services for COVID-19 patients are influenced by structural and social factors related to income, employment and health inequality^(6)^. This is corroborated in Dahlgren and Whitehead’s model of Social Determinants of Health (SDH)^(7)^, which are subdivided into layers, ranging from individual characteristics to broad factors in the health-disease process. In this representation, the base corresponds to individual aspects, such as age, gender and genetic factors; the second layer represents behaviors and lifestyle; the third layer discusses community and support networks; the fourth layer includes factors related to living conditions, food supply and access to essential services; and the last layer is expressed by economic, social and environmental conditions.

The conditions that influence health are multiple, interactive and modifiable. During the COVID-19 pandemic, some SDH have intensified social and health inequities, such as the increase in the unemployment rate, causing severe economic difficulties for families. This crisis has had a direct impact on aspects related to housing, with an intensification of precarious housing and the risk of eviction, especially among black people and low-income populations who were already marginalized and experiencing severe housing precariousness^(8)^.

The challenges faced due to the pandemic in Brazil have been immense and aggravated by the precarious living and health conditions, especially among residents of the peripheries. The approach to caring for COVID-19 patients has been concomitant with other diseases, such as arboviruses, influenza, tuberculosis, AIDS and other non-communicable diseases and illnesses, increasing the difficulties in health actions^(9)^. Discussing the effectiveness of access can help to improve the quality of patient care, in order to favor better treatment, prognosis and monitoring of the population’s health. For the purposes of this study, access is understood to be the set of dimensions that act as an intermediary between seeking and entering the service^(10)^.

Given the comprehensive and multi-systemic nature attributed to the effectiveness of access to health services for COVID-19 patients, we sought to answer the question: is there an association between the social determinants of health of COVID-19 patients and the domains of the PCATool? Thus, the objective was to verify the association between the social determinants of health and the access of COVID-19 patients to health services, based on the domains of the modified PCATool-Brazil.

## METHOD

### Type of Study

Analytical, cross-sectional study with a quantitative approach, which followed the recommendations of the Strengthening the Reporting of Observational Studies in Epidemiology (STROBE).

### Location, Population and Sample Definition

This study was carried out in three northeastern Brazilian states: Ceará, Maranhão and Pernambuco. The population consisted of patients reported with COVID-19, identified on the e-SUS-Notifica virtual reporting platform (https://notifica.saude.gov.br/onboard) and the flu reporting platform SIVEP-GRIPE (sivepgripe.saude.gov.br/sivepgripe/login.html) and provided by the State Health Departments of the three states. These states were chosen due to the high prevalence of COVID-19 cases during the pandemic.

The sample calculation used the number of infected people from the COVID-19 monitoring panel in Brazil, with a total of 829,038 infected people in the three states, from February 2020 to January 2021^(11)^. The sample was calculated randomly, probabilistically, using the infinite sample calculation. The confidence level was set at 95% and the absolute sampling error was 3.5%, resulting in a sample of 784 participants. For possible loss of information, a further 23% was added, resulting in a sample of 968 patients.

### Selection Criteria

The study included patients over the age of 19, notified of COVID-19 from January 2021 to February 2022, with a diagnosis of COVID-19 confirmed by laboratory testing; and telephone contact in the e-SUS Notifica or SIVEP-GRIPE records. Individuals who were not seen at a public health service (UBS, UPA, hospital) or private health service (clinic and hospital) for COVID-19 treatment, who reported being unable to respond to the study instrument, who did not answer calls after three attempts or who died were excluded.

### Data Collection

Data collection took place from August 2021 to March 2022, with questionnaires sent via links distributed randomly and automatically by software developed and validated for monitoring patients with COVID-19 and other respiratory syndromes^(12)^. The data on patients reported with COVID-19 was entered into the software using Excel spreadsheets.

The link generated by the software was linked to a message inviting the patient to take part in the study, which also contained a brief explanation of the research and instructions for completing the questionnaires. On accessing the link, the participant was directed to the Informed Consent Form and, subsequently, to the sociodemographic data and SDH instrument; and the PCATool-adapted to COVID-19. If it wasn’t possible to contact them via the telephone messaging app or if the links weren’t returned, they were interviewed by telephone, with up to three attempts to contact them in order to be included in the study.

### Data Collection Instrument

Two instruments were used: the first included data on the individual, according to the SDH model proposed by Dahlgren and Whitehead^(8)^; the second was the Primary Care Assessment Tool (PCATool-Brazil), which is divided into essential and derived attributes, the essential ones being: access from the individual’s first contact with the health system (utilization and accessibility), longitudinality, coordination of care (Integration of care and Information system) and comprehensiveness (Services available and Services provided). And the derivatives: family orientation and community orientation^(10)^. For this study, the PCATool was adapted to assess the care of people with COVID-19, in health services, during the illness and recovery process. The original instrument has 87 items, 45 of which were excluded because they were not related to the evaluation of health services provided to COVID-19 patients; and 16 items were added to cover aspects relevant to the evaluation of health services in the context of COVID-19, resulting in 58 items subdivided into nine domains. Validation was carried out with an adequate Content Validity Coefficient (CVC) and well evaluated in terms of clarity of language, practical relevance and theoretical relevance, with a CVC between 0.93 and 1.00. In addition, an overall Cronbach’s alpha of 0.868 was obtained, demonstrating the instrument’s reliability.

### Data Analysis and Processing

To analyze the PCATool-Brazil instrument, a value was assigned to each alternative on the Likert scale: “certainly yes”, with a value of 4; “probably yes”, with a value of 3; “probably not”, with a value of 2; “certainly not”, with a value of 1; and “don’t know/don’t remember”, with a value of 9. Each attribute assessed was calculated as the arithmetic mean of the item response values^(13)^.

It is worth noting that some items (B2, C7, C8, D7, D10) have a reverse value with the items “certainly yes”, with a value of 1; “probably yes”, with a value of 2; “probably no”, with a value of 3; “certainly no”, with a value of 4. Another point to note is the modification of the answers “don’t know/don’t remember”, with a value of 9, to a value of 2, “probably not”, if the sum of the answers was less than 50% of the total number of items in a component. This transformation is necessary in order to negatively score some characteristics of the health service that are not known by the interviewee. The scores were transformed into a scale of zero to 10, as established by the Instrument Evaluation Manual, and classified as high (score ≥ 6.6) and low (score < 6.6)^(13)^.

In addition, the data was recorded in Microsoft office Excel 2013 spreadsheets and transferred and analyzed using the Statistical Package for the Social Sciences (SPSS), version 20.0. The age range was grouped into 20 to 39 years (young adult), 40 to 59 years (adult) and > 60 years (elderly). The Kolmogorov-Smirnov normality test was used for inferential analysis. Pearson’s chi-squared test was applied to check for differences between the high and low scores for each domain, according to the patients’ characteristics, with p-values <0.05 being considered a significant difference.

To control for effect-modifying variables, it was considered that confirmation of COVID-19 by laboratory test is an important method for differentiating it from other types of respiratory disease. Therefore, the sample was randomized to avoid confounding variables, such as a disproportionate sample or selection bias.

### Ethical Aspects

This article was cleared by the Research Ethics Committee, according to opinion N0 4.278.495. The research complies with Resolution 466 of December 12, 2012, and the Informed Consent Form was adopted.

## RESULTS

The sample consisted of 968 people from three northeastern Brazilian states (Ceará, Maranhão and Pernambuco). Table 1 shows the sociodemographic data of the participants. The majority were female (N = 580; 59.9%); aged between 20 and 94 years, predominantly over 39 years (N = 493; 50.9%), with an average age of 42.7 ± 15.3 years; non-white skin color (N = 699; 72.3%); with a BMI in the overweight or obese range (N = 563; 66.8%); married/stable union (N = 500; 51.7%); schooling up to high school (N = 541; 55.9%); family income of up to three minimum wages (N = 450; 46.5%), with an average of 3. 330 ± 5,467 reais per month. It is worth noting that some participants (N = 377; 38.9%) did not answer the item referring to financial income.

**Table 1:** Distribution of patients according to PCATool domains and social determinants of health, in Ceará, Maranhão and Pernambuco, Brazil, 2022.

PCAToolDomains	UT		AC		LO		CA. S		INF. S		CA. A		CA. D		FAM.O		COM. O		T
	L <6,6	H ≥6,6	L <6,6	H ≥6,6	L <6,6	H ≥6,6	L <6,6	H ≥6,6	L <6,6	H ≥6,6	L <6,6	H ≥6,6	L <6,6	H ≥6,6	L <6,6	H ≥6,6	L <6,6	H ≥6,6	
Social Determinants for Health	n(%)	n(%)	n(%)	n(%)	n(%)	n(%)	n(%)	n(%)	n(%)	n(%)	n(%)	n(%)	n(%)	n(%)	n(%)	n(%)	n(%)	n(%)	n(%)
**1** ^st^ **layer – Age, sex and hereditary factors**																			
Sex – P-value*	0,846		0,904		0,113		0,482		0,912		0,681		0,576		0,325		0,761		
Women	490 (84,5)	90 (15,5)	421 (72,6)	159 (27,4)	369 (63,6)	211 (36,4)	494 (85,2)	86 (14,8)	520 (89,7)	60 (10,3)	503 (86,7)	77 (13,3)	372 (64,1)	208 (35,9)	365 (62,9)	215 (37,1)	512 (88,3)	68 (11,7)	580 (59,9)
Male	326 (84,0)	62 (16,0)	283 (72,9)	105 (27,1)	266 (68,6)	122 (31,4)	324 (83,5)	64 (16,5)	347 (89,4)	41 (10,6)	340 (87,6)	48 (12,4)	242 (62,4)	146 (37,6)	232 (59,8)	156 (40,2)	340 (87,6)	48 (12,4)	388 (40,1)
Age (years) – P-value*	**0,012**		**0,035**		0,411		0,369		**0,011**		**0,013**		0,826		0,077		**0,057**		
20 – 39	391 (82,3)	84 (17,3)	347 (73,1)	128 (26,9)	323 (68,0)	152 (32,0)	413 (86,9)	62 (13,1)	416 (87,6)	59 (12,4)	401 (84,4)	74 (15,6)	306 (64,4)	169 (35,6)	303 (63,8)	172 (36,2)	428 (90,1)	47 (9,9)	475 (49,1)
40 – 59	277 (82,9)	57 (17,1)	234 (70,1)	100 (29,9)	209 (62,6)	125 (37,4)	274 (82,0)	60 (18,0)	298 (89,2)	36 (10,8)	292 (87,4)	42 (12,6)	212 (63,5)	122 (36,5)	212 (63,5)	122 (36,5)	280 (83,8)	54 (16,2)	334 (34,5)
> 60	148 (93,1)	11 (6,9)	123 (77,4)	36 (22,6)	103 (64,8)	56 (35,2)	131 (82,4)	28 (17,6)	153 (96,2)	6 (3,8)	150 (94,3)	9 (5,7)	96 (60,4)	63 (39,6)	82 (51,6)	77 (48,4)	144 (90,6)	15 (9,4)	159 (16,4)
Skin color P-value*	**0,004**		0,165		0,173		0,913		**0,011**		**0,002**		0,823		0,472		0,642		
Brown	445 (83,3)	89 (16,7)	386 (72,3)	148 (27,7)	348 (65,2)	186 (34,8)	454 (85,0)	80 (15,0)	478 (89,5)	56 (10,5)	461 (86,3)	73 (13,7)	344 (64,4)	190 (35,6)	338 (63,3)	196 (36,7)	466 (87,3)	68 (12,7)	534 (55,1)
White	217 (80,7)	52 (19,3)	188 (69,9)	81 (30,1)	169 (62,8)	100 (37,2)	224 (83,3)	45 (16,7)	231 (85,9)	38 (14,1)	224 (83,3)	45 (16,7)	167 (62,1)	102 (37,9)	157 (58,4)	112 (41,6)	238 (88,5)	31 (11,5)	269 (27,8)
Black	133 (93,7)	9 (6,3)	110 (77,5)	32 (22,5)	99 (69,7)	43 (30,3)	120 (84,5)	22 (15,5)	135 (95,1)	7 (4,9)	136 (95,8)	6 (4,2)	90 (63,4)	52 (36,6)	86 (60,6)	56 (39,4)	126 (88,7)	16 (11,3)	142 (14,7)
Yellow	21 (91,3)	2 (8,7)	20 (87,0)	3 (13,0)	19 (82,6)	4 (17,4)	20 (87,0)	3 (13,0)	23 (100,0)	0 (0,0)	22 (95,7)	1 (4,3)	13 (56,5)	10 (43,5)	16 (69,6)	7 (30,4)	22 (95,7)	1 (4,3)	23 (2,4)
**2** ^nd^ **layer – Individual lifestyles**																			
BMI – P-value*	**<0,001**		**<0,001**		0,683		**0,033**		**0,004**		**<0,001**		**0,023**		0,558		0,406		
Low	35 (89,7)	4 (10,3)	32 (82,1)	7 (17,9)	26 (66,7)	13 (33,3)	36 (92,3)	3 (7,7)	35 (89,7)	4 (10,3)	38 (97,4)	1 (2,6)	34 (87,2)	5 (12,8)	22 (56,4)	17 (43,6)	35 (89,7)	4 (10,3)	39 (4,6)
Eutrophic	209 (86,7)	32 (13,3)	177 (73,4)	64 (26,6)	153 (63,5)	88 (36,5)	215 (89,2)	26 (10,8)	217 (90,0)	24 (10,0)	212 (88,0)	29 (12,0)	153 (63,5)	88 (36,5)	147 (61,0)	94 (39,0)	219 (90,9)	22 (9,1)	241 (28,6)
Overweight	283 (85,2)	49 (14,8)	253 (76,2)	79 (23,8)	217 (65,4)	115 (34,6)	278 (83,7)	54 (16,3)	303 (91,3)	29 (8,7)	291 (87,7)	41 (12,3)	210 (63,3)	122 (36,7)	202 (60,8)	130 (39,2)	288 (86,7)	44 (13,3)	332 (39,4)
Obesity I	117 (74,1)	41 (25,9)	95 (60,1)	63 (39,9)	96 (60,8)	62 (39,2)	129 (81,6)	29 (18,4)	131 (82,9)	27 (17,1)	124 (78,5)	34 (21,5)	98 (62,0)	60 (38,0)	100 (63,3)	58 (36,7)	137 (86,7)	21 (13,3)	158 (18,7)
Obesity II	48 (65,8)	25 (34,2)	43 (58,9)	30 (41,1)	42 (57,5)	31 (42,5)	56 (76,7)	17 (23,3)	57 (78,1)	16 (21,9)	54 (74,0)	19 (26,0)	41 (56,2)	32 (43,8)	38 (52,1)	35 (47,9)	61 (83,6)	12 (16,4)	73 (8,7)
**3** ^rd^ **layer – Social and community networks**																			
Marital status – P-value*	0,658		0,644		0,260		0 ,369		0,731		0,566		0,521		0,656		0,940		
Single	299 (84,7)	54 (15,3)	256 (72,5)	97 (27,5)	247 (70,0)	106 (30,0)	300 (85,0)	53 (15,0)	317 (89,8)	36 (10,2)	306 (86,7)	47 (13,3)	233 (66,0)	120 (34,0)	213 (60,3)	140 (39,7)	309 (87,5)	44 (12,5)	353 (36,5)
Married/Civil union	421 (84,2)	79 (15,8)	369 (73,8)	131 (26,2)	318 (63,6)	182 (36,4)	428 (85,6)	72 (14,4)	447 (89,4)	53 (10,6)	438 (87,6)	62 (12,4)	307 (61,4)	193 (38,6)	313 (62,6)	187 (37,4)	442 (88,4)	58 (11,6)	500 (51,7)
Separated	60 (83,3)	12 (16,7)	47 (65,3)	25 (34,7)	45 (62,5)	27 (37,5)	57 (79,2)	15 (20,8)	63 (87,5)	9 (12,5)	61 (84,7)	11 (15,3)	49 (68,1)	23 (31,9)	48 (66,7)	24 (33,3)	62 (86,1)	10 (13,9)	72 (7,4)
Widowed	36 (83,7)	71 (6,3)	32 (74,4)	11 (25,6)	25 (58,1)	18 (41,9)	33 (76,7)	10 (23,3)	40 (93,0)	3 (7,0)	38 (88,4)	5 (11,6)	25 (58,1)	18 (41,9)	23 (53,5)	20 (46,5)	39 (90,7)	4 (9,3)	43 (4,4)
**4** ^th^ **layer – Living and working conditions**																			
Schooling – P-value*	**<0,001**		**0,002**		0,329		**0,001**		**<0,001**		**<0,001**		0,137		0,361		**0,010**		
Illiterate	27 (81,8)	6 (18,2)	21 (63,6)	12 (36,4)	19 (57,6)	14 (42,4)	23 (69,7)	10 (30,3)	28 (84,8)	51 (5,2)	28 (84,8)	5 (5,2)	21 (63,6)	12 (36,4)	16 (48,5)	17 (51,5)	28 (84,8)	5 (15,2)	33 (3,4)
Elementary school	128 (89,5)	15 (10,5)	114 (79,7)	29 (20,3)	101 (70,6)	42 (29,4)	121 (84,6)	22 (15,4)	135 (94,4)	8 (5,6)	132 (92,3)	11 (7,7)	89 (62,2)	54 (37,8)	83 (58,0)	60 (42,0)	126 (88,1)	17 (11,9)	143 (14,8)
High school	362 (91,0)	36 (9,0)	302 (75,9)	96 (24,1)	265 (66,6)	133 (33,4)	355 (89,2)	43 (10,8)	374 (94,0)	24 (6,0)	371 (93,2)	27 (6,8)	271 (68,1)	127 (31,9)	255 (64,1)	143 (35,9)	360 (90,5)	38 (9,5)	398 (41,1)
Higher education	247 (79,9)	62 (20,1)	218 (70,6)	91 (29,4)	192 (62,1)	117 (37,9)	254 (82,2)	55 (17,8)	270 (87,4)	39 (12,6)	258 (83,5)	51 (16,5)	182 (58,9)	127 (41,1)	189 (61,2)	120 (38,8)	273 (88,3)	36 (11,7)	309 (31,9)
Post graduate	52 (61,2)	33 (38,8)	49 (57,6)	36 (42,4)	58 (68,2)	27 (31,8)	65 (76,5)	20 (23,5)	60 (70,6)	25 (29,4)	54 (63,5)	31 (36,5)	51 (60,0)	34 (40,0)	54 (63,5)	31 (36,5)	65 (76,5)	20 (23,5)	85 (8,8)
Healthcare service – P-value*	**<0,001**		**<0,001**		0,989		**<0,001**		**<0,001**		**<0,001**		0,178		0,083		0,662		
Public	791 (86,9)	119 (13,1)	679 (74,6)	231 (25,4)	597 (65,6)	313 (34,4)	771 (84,7)	139 (15,3)	825 (90,7)	85 (9,3)	809 (88,9)	101 (11,1)	582 (64,0)	328 (36,0)	555 (61,0)	355 (39,0)	802 (88,1)	108 (11,9)	910 (94,0)
Private	25 (43,1)	33 (56,9)	25 (43,1)	33 (56,9)	38 (65,5)	20 (34,5)	47 (81,0)	11 (19,0)	42 (72,4)	16 (27,6)	34 (58,6)	24 (41,4)	32 (55,2)	26 (44,8)	42 (72,4)	16 (27,6)	50 (86,2)	8 (13,8)	58 (6,0)
**5** ^th^ **layer – General socio-economic, cultural and environmental conditions**																			
**Income – P-value (in minimum wages)**	0,127		**0,040**		0,751		0,485		0,646		**0,016**		0,797		0,745		0,837		
≤ 1	182 (82,7)	38 (17,3)	166 (75,5)	54 (24,5)	147 (66,8)	73 (33,2)	177 (80,5)	43 (19,5)	194 (88,2)	26 (11,8)	189 (85,9)	31 (14,1)	143 (65,0)	77 (35,0)	133 (60,5)	87 (39,5)	186 (84,5)	34 (15,5)	220 (22,7)
2 – 3	177 (77,0)	53 (23,0)	155 (67,4)	75 (32,6)	143 (62,2)	87 (37,8)	186 (80,9)	44 (19,1)	194 (84,3)	36 (15,7)	188 (81,7)	42 (18,3)	139 (60,4)	91 (39,6)	147 (63,9)	83 (36,1)	201 (87,4)	29 (12,6)	230 (23,8)
4 – 5	66 (80,5)	16 (19,5)	59 (72,0)	23 (28,0)	54 (65,9)	28 (34,1)	68 (82,9)	14 (17,1)	69 (84,1)	13 (15,9)	67 (81,7)	15 (18,3)	52 (63,4)	30 (36,6)	54 (65,9)	28 (34,1)	71 (86,6)	11 (13,4)	82 (8,5)
> 6	41 (69,5)	18 (30,5)	34 (57,6)	25 (42,4)	37 (62,7)	22 (37,3)	43 (72,9)	16 (27,1)	50 (84,7)	91 (5,3)	40 (67,8)	19 (32,2)	37 (62,7)	22 (37,3)	35 (59,3)	24 (40,7)	50 (84,7)	9 (15,3)	59 (6,1)

* Chi-square Test.

Captions: UT: Utilization; AC: Accessibility; LO: Longitudinality; CA. S: Care System; I. S: Information system; CA. A: Care Available; CA. D: Care delivered; FAM. O: Family orientation; COM. O: Community orientation; L: Low score; H: High score; T: Total; BMI: Body Mass Index.

Table 1 also shows the domains of the PCATool, with sociodemographic characteristics and social determinants of health. The results showed that, in the first tier of the SDH, there was no significant difference (p > 0.05) between the gender variable and the scores in the nine domains of the PCATool. Age showed a significant difference in the domains of use (p = 0.012), accessibility (p = 0.035), information system (p = 0.011), services available (p = 0.013) and community orientation (p = 0.057). There was a predominance of low scores (<6.6) in these domains at the extremes of age, considering the 20–29 and 60–94 age groups.

In the skin color variable, there was a significant difference in the domains of use (p = 0.004), information system (p = 0.011) and available services (p = 0.002), with a predominant low score for non-white people, considering brown, black and yellow.

The second layer of SDH, which assessed BMI, showed significant differences in the domains of use (p < 0.001), accessibility (p < 0.001), care system (p = 0.003), information system (p = 0.004), services available (p < 0.001) and services provided (p = 0.023). All the domains showed higher percentages of low scores for people with a low BMI. On the other hand, people with grade II obesity showed a higher percentage of high PCATool scores in all the domains, when compared to the other BMI classifications.

As shown in the third layer of the SDH, social and community networks, marital status showed no significant difference in any domain of the PCATool.

The fourth layer of the SDH, referring to living and working conditions, showed a significant difference in the schooling variable in the domains of use (p < 0.001), accessibility (p = 0.002), care system (p = 0.001), information system (p < 0.001) and services available (p < 0.001) and community orientation (p = 0.001), with people with a low level of schooling predominating in the low scores. This layer also analyzed the type of service (public or private) where the patient was seen, where p < 0.001 was found for the domains utilization, accessibility, care system, information system and available services, where the highest percentages of low scores were found in public health services.

In the fifth layer of the SDH, patients with an income of up to one minimum wage had higher percentages of low scores in the accessibility (p = 0.04) and available services (p = 0.016) domains, whose domains showed a significant difference in the inferential analysis.

## DISCUSSION

The descriptive analysis of the social determinants of health showed a predominance of females, ages over 39, non-white skin color and high school education. These data corroborate a study carried out in Rio de Janeiro, Brazil, which showed a higher percentage of adult and elderly patients with COVID-19^(14)^.

The inferential analyses highlighted people aged between 60 and 94 with a high percentage in the low scores for use of services, information system and available services, demonstrating the difficulty of the elderly population in getting care in health services, withholding information contained in medical records and deficits in available services. In the elderly population, various factors can contribute to hindering access to and use of health services, such as socio-economic aspects, difficulties in getting around, the absence of a partner and frailty^(15)^.

However, the elderly had lower percentages of low scores in relation to the family orientation domain, showing that they received more clarification/guidance compared to the other age groups. Preventive measures for the elderly should be the same as those indicated for people in all life cycles, but with increased guidance for family members and caregivers^(16)^. Similarly, there was significance between age and the accessibility and community orientation domains, with people aged between 20 and 29 showing low scores in both domains.

In the analysis of skin color, lower scores were found for black, brown and yellow people, demonstrating difficulty in using health services and fewer services available to black populations. This result may be related to the fact that the black population has greater difficulties in gaining access to health services and equipment and faces various problems, such as precarious housing, homes without water supply and/or sewage disposal, lower income and higher rates of food insecurity^(17)^. Marginalized populations have also shown various disparities in terms of exposure, susceptibility and access to services during the pandemic, with data showing that black or Latino people have reported lower levels of access to COVID-19 tests, medical services and the use of telehealth for mental health monitoring^(18)^.

In terms of BMI, the domains that showed statistical differences had higher percentages of high scores for people with type II obesity and lower percentages of low scores for people with a BMI below the appropriate level. This can be explained by the fact that obesity is a risk factor for complications of COVID-19 and has priority access to health services in the Northeast of Brazil. In addition, during the pandemic, food security was impacted by various effects/events, such as rising food prices, problems in food production, availability and marketing, reduced wages and unemployment, causing greater social inequalities and less access to healthy food for the most vulnerable social classes^(19)^. In the United States, the economic crisis and unemployment have accelerated levels of food insecurity, with 45% of Latino families in Los Angeles having difficulty in getting access to food^(20)^.

In the fourth tier of SDH, schooling showed a significant difference in relation to six domains of the PCATool, being influenced by elementary and high school education, which showed low scores. Schooling plays an important role in various contexts and situations, and the unequal supply of educational activities has an impact on occupations and social mobility^(17)^.

The first care service for COVID-19 patients showed predominantly low scores for people treated in Primary Health Care Units and high scores for those treated in hospitals and testing units. The Primary Care service in Brazil has some adversities that can influence its performance, such as patients seeking other services to solve the demands of the Basic Health Unit; difficulty in managing chronic and communicable diseases; a lack of training incentives; and a shortage of material and financial resources^(21)^.

Similarly to the aspects mentioned above, care provided in public or private services was also statistically significant, with higher percentages of low scores in public health services. Studies that have analyzed health care in these two types of services have shown some factors that can affect or enhance access to these services, such as priority/rapidity in care and a greater number of referrals to private services, infrastructure problems, lower travel costs and a greater number of visits to the public network^(22,23)^. In addition, the COVID-19 pandemic has posed a number of challenges for health systems, mainly due to the increase in costs and the number of beds, making it necessary to review management models, access, processes and funding policies^(24)^.

Related to income, it was noted that in the domains of accessibility and services available, people with an income of up to one minimum wage had a predominance of low scores, demonstrating greater difficulty in access and fewer services available to people on low incomes. A study carried out in Shanghai, China, evaluating equity in the accessibility of health services, showed that high-income families had better access to health services than low-income families^(25)^. Another factor influencing reduced access to health services is the scarcity of social support networks operating within health services and aimed at vulnerable patients^(26)^.

At the same time, low-income populations were considered more vulnerable to SARS-CoV-2 infection during the pandemic, due to various factors, such as greater use of public transport services, a greater number of residents in the same household, limited access to basic sanitation and health care, and difficulty in maintaining social isolation, due to the need to maintain income or employment^(27)^.

Social aspects such as income, access to education, living and working conditions can shape health inequalities, which have been strongly evident during the COVID-19 pandemic^(28)^. When conducting an in-depth discussion on the relationships that existed during the COVID-19 pandemic, some researchers considered it a syndemic, due to its coexistence with other diseases and the potentialization of this relationship with SDH, such as the social, economic and environmental context of populations^(29,30)^.

The study brought an unprecedented approach, as it correlated and identified the social determinants of health related to access to health services during the pandemic, which through this, provide knowledge and strategies for improving health service care. Knowledge of the SDH allows for the establishment of actions that involve society as a whole to consciously adopt precautionary measures in the face of COVID-19, considering changes in individual and collective behavior in the pandemic scenario, given that the economic, social and health impacts in Brazil depend on the collaborative effort of everyone, public authorities, families and citizens. Furthermore, the results can guide actions, funding and the development of health policies aimed at patients with COVID-19 and other diseases in an epidemic/pandemic situation.

The study’s limitations include the online collection method, which made it difficult to approach people who do not have access to communication technologies, such as cell phones or the internet; obtaining data made available by the health department, whose spreadsheets contained missing data and incorrect telephone contact numbers; and the different sample size between the three states, with Maranhão having a higher number of acceptances and responses compared to Pernambuco and Ceará.

## CONCLUSION

This study assessed the associations between the social determinants of health of COVID-19 patients and access to and use of health services in three states in the Northeast of Brazil, using the PCATool adapted to the context of the COVID-19 pandemic.

In the analysis of the social determinants of health, it was found that access was directly related to various factors, such as individual aspects, lifestyle, support and community networks, living, working, socioeconomic, cultural and environmental conditions in the three states of Northeast Brazil. In view of these results, health managers and the governments of these states can direct actions and funding in the health area towards the most critical points, such as integration of care and community orientation. It is suggested that other studies should be carried out to compare the pre- and post-pandemic scenario, as well as studying these aspects in other Brazilian regions.

## References

[1] Zhu N, Zhang D, Wang W, Li X, Yang B, Song J (2020). China Novel Coronavirus Investigating and Research Team. A novel coronavirus from patients with pneumonia in China, 2019. N Engl J Med.

[2] Shafaghi AH, Rokhsar Talabazar F, Koşar A, Ghorbani M (2020). On the effect of the respiratory droplet generation condition on COVID-19 transmission. Fluids.

[3] Brasil (2020). Doença pelo Coronavírus COVID-19 [Internet].

[4] World Health Organization (2023). Coronavirus (COVID-19) Dashboard [Internet].

[5] Shamasunder S, Holmes SM, Goronga T, Carrasco H, Katz E, Frankfurter R (2020). COVID-19 reveals weak health systems by design: why we must re-make global health in this historic moment. Glob Public Health.

[6] Abedi V, Olulana O, Avula V, Chaudhary D, Khan A, Shahjouei S (2021). Racial, economic, and health inequality and COVID-19 infection in the United States. J Racial Ethn Health Disparities.

[7] Dahlgren G, Whitehead M (1991). Policies and strategies to promote social equity in health.

[8] Benfer EA, Vlahov D, Long MY, Walker-Wells E, Pottenger JL, Gonsalves G (2021). Eviction, health inequity, and the spread of COVID-19: housing policy as a primary pandemic mitigation strategy. J Urban Health.

[9] Oliveira WKD, Duarte E, França GVAD, Garcia LP (2020). How Brazil can hold back COVID-19. Epidemiol Serv Saude.

[10] Penchansky R, Thomas JW (1981). The concept of access: definition and relationship to consumer satisfaction. Med Care.

[11] Brasil (2021). Coronavírus/Brasil [Internet].

[12] Fontenele MGM (2021). Desenvolvimento e avaliação de software para monitoramento de pacientes com Covid-19 e outras síndromes respiratórias [dissertação].

[13] Brasil (2020). Ministério da Saúde. Manual do Instrumento de Avaliação da Atenção Primária à Saúde: PCATool-Brasil [Internet].

[14] Alves AT, Raposo LM, Nobre FF (2022). Spatial analysis of the sociodemographic characteristics, comorbidities, hospitalization, signs, and symptoms among hospitalized coronavirus disease 2019 cases in the state of Rio De Janeiro, Brazil. Int J Health Serv.

[15] Tavares DMDS, Oliveira NGN, Marchiori GF, Marmo FAD, Jesus DAD (2021). Access to and use of the health services among community older adults. Cogitare Enfermagem.

[16] Souza JHA (2020). Isolamento social versus qualidade de vida dos idosos: um olhar multiprofissional frente à pandemia do Covid-19. Rev Pub Saúde.

[17] Instituto Brasileiro de Geografia e Estatística (2020). Informativo IBGE sobre desigualdades sociais por cor ou raça no Brasil [Internet].

[18] Ruprecht MM, Wang X, Johnson AK, Xu J, Felt D, Ihenacho S (2021). Evidence of social and structural COVID-19 disparities by sexual orientation, gender identity, and race/ethnicity in an urban environment. J Urban Health.

[19] Silva RDCR, Pereira M, Campello T, Aragão É, Guimarães JMDM, Ferreira AJ (2020). Covid-19 pandemic implications for food and nutrition security in Brazil. Cien Saude Colet.

[20] Payán DD, Díaz Rios LK, Ramírez AS, De Trinidad Young ME (2021). Structural barriers influencing food insecurity, malnutrition, and health among Latinas during and after COVID-19: considerations and recommendations. J Acad Nutr Diet.

[21] Geremia DS (2020). Atenção Primária à Saúde em alerta: desafios da continuidade do modelo assistencial. Physis.

[22] Hasenclever L, Miranda C, Chaves G, Peixoto ALA, Mattos LV, Viana JDS (2021). Controversial aspects of the concept of health needs and their impact on the accessibility of medicine and health services. Cien Saude Colet.

[23] Almeida APSC, Nunes BP, Duro SMS, Lima RDCD, Facchini LA (2020). Lack of access and the trajectory of healthcare use by elderly Brazilians. Cien Saude Colet.

[24] Brancalion FNM, Lima AFC (2022). Process-based management aimed at improving health care and financial results. Rev Esc Enferm USP.

[25] Jin T, Cheng L, Wang K, Cao J, Huang H, Witlox F (2022). Examining equity in accessibility to multi-tier healthcare services across different income households using estimated travel time. Transp Policy.

[26] Farias CML, Moraes L, Degli Esposti CD, Santos No ET (2020). Absenteísmo de usuários: barreiras e determinantes no acesso aos serviços de saúde. Rev Bras Med Fam Comunidade.

[27] Mendonça MHM, Silva AGS, Cunha CLF, Latgé PK (2020). A pandemia COVID-19 no Brasil: ecos e reflexos nas comunidades periféricas. APS Rev.

[28] Silva FAJ, Peres AM, Lourenço RG, Souza MAR, Figueiredo KC, Camargo CL (2023). Primary Healthcare of black immigrants during the COVID-19 pandemic. Rev Esc Enferm USP.

[29] Bispo JP, Santos DB (2021). COVID-19 as a syndemic: a theoretical model and foundations for a comprehensive approach in health. Cad Saude Publica.

[30] Caron RM, Adegboye ARA (2021). COVID-19: a syndemic requiring an integrated approach for marginalized populations. Front Public Health.

